# Gut Microbiota and Inflammation

**DOI:** 10.3390/nu3060637

**Published:** 2011-06-03

**Authors:** Asa Hakansson, Goran Molin

**Affiliations:** Food Hygiene, Division of Applied Nutrition, Department of Food Technology, Engineering and Nutrition, Lund University, PO Box 124, SE-22100 Lund, Sweden; Email: asa.hakansson@appliednutrition.lth.se

**Keywords:** probiotics, inflammation, gut microbiota

## Abstract

Systemic and local inflammation in relation to the resident microbiota of the human gastro-intestinal (GI) tract and administration of probiotics are the main themes of the present review. The dominating taxa of the human GI tract and their potential for aggravating or suppressing inflammation are described. The review focuses on human trials with probiotics and does not include *in vitro* studies and animal experimental models. The applications of probiotics considered are systemic immune-modulation, the metabolic syndrome, liver injury, inflammatory bowel disease, colorectal cancer and radiation-induced enteritis. When the major genomic differences between different types of probiotics are taken into account, it is to be expected that the human body can respond differently to the different species and strains of probiotics. This fact is often neglected in discussions of the outcome of clinical trials with probiotics.

## 1. Inflammation

Inflammation is a defence reaction of the body against injury. The word inflammation originates from the Latin word “inflammatio” which means fire, and traditionally inflammation is characterised by redness, swelling, pain, heat and impaired body functions. Redness and heat are caused by increased blood flow, swelling by accumulation of fluid, and pain by the swelling, but also by release of compounds giving rise to nerve signals. Impaired functions are caused by different reasons but, in a certain analogy to fire, inflammation is devastating in order to clear away harmful agents and therefore prepare the ground for re-growth (healing). 

Inflammation can be triggered off by both internal and external factors. Powerful triggers for inflammation are the presence of microorganisms in sites where they do not belong. Microorganisms contain structures alien to the body. Bacteria and fungi, for example, have cell walls in contrast to human cells that lack these structures, and viruses have unique forms of DNA and RNA. Cells and molecules involved in the inflammatory defence system react immediately against these foreign elements; they are danger signals to the body. In addition, injuries to body tissue and cells trigger inflammation. When the body cells are damaged, compounds that are normally hidden within the cells are released and work as endogenous danger signals. All forms of immune reactions will lead to activation of the inflammatory defence system. Consequently, inflammation can be started by infections, decomposition of body tissue by trauma (for example, due to surgery or accidents) and autoimmunity or allergy. In autoimmunity the specific immune system attacks body cells and tissue and releases the inflammation, and in allergy the inflammation is provoked by the specific immune system being activated against different types of harmless compounds in the environment, e.g., food and pollen. 

The process of inflammation is initiated by cells already present in the tissue, e.g., resident macrophages, dendritic cells and mast cells. Danger signals trigger these cells into activation, and inflammatory mediators are released, which starts the process responsible for the clinical signs of inflammation. The process of inflammation involves four stages:

(*i*) Blood vessels widen, resulting in increased blood flow (causing the redness and increased heat);
(*ii*) Permeability of the blood vessels is increased, which results in an outflow of fluid and plasma proteins into the tissue, manifesting itself as swelling;
(*iii*) White blood cells are recruited from the blood circulation to the tissue;
(*iv*) The metabolism is adjusted, for example by increased levels of glucose in the blood, and symptoms such as fever, fatigue and loss of appetite can occur.

When the process of inflammation has been initiated, it will proceed along a certain course of events until the source of the inflammation has been erased and the healing process can start. However, if the cause of the inflammation cannot be eliminated, the inflammation will continue, and then it will often vary in intensity over time. 

In acute inflammation, there will be an accumulation of neutrophil granulocytes (neutrophils) in the inflamed tissue, while in chronic inflammation there will be an accumulation of lymphocytes, macrophages and plasma cells in the tissue and also infiltrating connection tissue. In an allergic reaction, however, there will be a rapid accumulation of eosinophil granulocytes (eosinophils) and T-lymphocytes, and sometimes also neutrophils. A representative example of a situation leading to acute inflammation is a bacterial infection, but cell death at infarct of the heart or decomposition of cancer tumours will also lead to acute inflammation. Typical causes of chronic inflammation are infections with intracellular bacteria, autoimmune diseases, contact allergy and reactions against foreign elements [1].

In an acute inflammatory response, the concentration of acute phase proteins such as C reactive protein (CRC) and serum amyloid A protein (SAA) can increase steeply and rise to 10,000-fold above base-line [2]. However, different markers for acute inflammation can also be monitored more closely where more subtle and inflexible systemic alterations are taken into consideration. This type of slight elevation from the norm can be called “low-grade inflammation”, or “subclinical inflammation”. Consequently, in this type of condition the sharp short-term fluctuations of inflammatory markers are ignored; instead, long-term systemic concentrations of the markers are considered, especially if they correlate with more obvious risk factors such as, for example, blood cholesterol and blood pressure. Low-grade systemic inflammation, mainly characterised by increased CRP, is associated with an increased risk of cardiovascular disease [3], and obese individuals have higher CRP levels than subjects of normal weight [4,5].

The intestinal immune system has developed a tightly regulated control to optimise the protection against pathogens, while at the same time avoiding unnecessary immune activity. The intestine is a primary site of foreign antigen encounter and it is associated with several types of lymphoid organs collectively referred to as gut-associated lymphoid tissue (GALT). GALT is the largest collection of lymphoid tissues in the body and consists of organised lymphoid tissues comprising mesenteric lymph nodes, Payer´s patches, isolated lymphoid follicles, and cryptopatches, as well as diffusely scattered lymphocytes and dendritic cells in the lamina propria and intestinal epithelium [6,7,8]. Some of them, such as Payer’s patches and the isolated lymphoid follicles, are within the mucosa itself. In addition, intestinal lymph drains into the mesenteric lymph nodes, which constitute a key checkpoint to determine the anatomical location of tolerogenic or inflammatory responses [9].

In inflammation, macrophages have three major functions, namely: (*i*) antigen presentation, (*ii*) phagocytosis and (*iii*) immune-modulation through production of various cytokines and growth factors. Monocytes/macrophages produce a wide range of biologically active molecules participating in both beneficial and detrimental outcomes of inflammatory reactions. They are also able to phagocytose and destroy infectious agents. Therefore, monocytes/macrophages play a critical role in initiation, maintenance, and resolution of inflammation [10,11]. Macrophages form varying phenotypes depending on what signals they encounter [12]. Different subsets of macrophages express different patterns of chemokines, surface markers and metabolic enzymes. Classically activated macrophages (proinflammatory M1) induced by proinflammatory mediators, such as lipopolysaccharide (LPS), IL-1β and IFN-γ, produce proinflammatory cytokines (TNF-α, IFN-γ, IL-6 and IL-12) and generate reactive oxygen species [13,14]. In contrast, M2 macrophages, alternatively activated by exposure to, for example, IL-4, IL-13 and IL-10, produce less proinflammatory cytokines than M1, and instead produce more components signalling anti-inflammation, for example, IL-10, TGF-β and IL-1 receptor antagonist [14]. M2 macrophages are believed to participate in the blockade of inflammatory responses and promotion of tissue repair and type II immunity [15]. Consequently, different macrophage subsets have different roles in both inflammation and modulation of the immune response or tolerance.

Microbial colonisation of the GI tract affects the composition of GALT. Immediately after exposure to luminal microorganisms, the number of intraepithelial lymphocytes expands greatly [16,17], germinal centres with immunoglobulin-producing cells arise rapidly in follicles and in the lamina propria [18], and concentrations of immunoglobulin increase substantially in serum [19].

There is a complex relationship between the intestinal immune system and the resident GI microbiota and it is crucial for the epithelial cells and the mucosal immune system to distinguish between pathogenic and non-pathogenic agents. Intestinal epithelial cells are capable of detecting bacterial antigens and initiating and regulating both innate and adaptive immune responses. Signals from bacteria can be transmitted to adjacent immune cells such as macrophages, dendritic cells and lymphocytes through molecules expressed on the epithelial cell surface, such as major histo-compatibility complex I and II molecules and Toll-like receptors (TLRs) [20,21]. TLRs alert the immune system to the presence of highly conserved microbial antigens often termed “pathogen-associated molecular patterns” (PAMPs) present on most microorganisms. Examples of PAMPs include lipopolysaccharides (LPS), peptidoglycan, flagellin, and microbial nucleic acids. TRLs are so named because of their similarity to a receptor first identified in the fruit fly *Drosophila melanogaster*, a protein coded by the *Toll*-gene (“toll” means fantastic in German). At least ten types of human TLRs are known. In healthy adults, TLRs are expressed in most tissues, including myelomonocytic cells, dendritic cells and endothelial and epithelial cells. Interaction of TLRs and bacterial molecular patterns results in activation of a complex intracellular signalling cascade, up-regulation of inflammatory genes, production of pro-inflammatory cytokines and interferons, and recruitment of myeloid cells. It also stimulates expression of co-stimulatory molecules required to induce an adaptive immune response of antigen presenting cells [22]. Epithelium in, for example, colon shows a comparably high level of expression of TLR3, TLR4, TLR5, and TLR7, with TLR3 being the most abundant [23], while cervical and vaginal epithelial cells have a higher expression of TLR1, TLR2, TLR3, TLR5 and TLR6 [24]. TLR4 recognises lipopolysaccharide (LPS) [25,26], a constituent of the cell wall of Gram-negative bacteria, while TLR2 reacts with a wider spectrum of bacterial products such as lipoproteins, peptidoglycans and lipoteichoic acid which can be found in both Gram-positive and Gram-negative bacteria [27,28].

Besides the TLRs there is another family of membrane-bound receptors for detection of proteins called NOD-like receptors or “nucleotide-binding domain, leucine-rich repeat containing” proteins (NLRs). The best characterised members are NOD1 and NOD2, but more than twenty different NLRs have been identified. NRLs are located in the cytoplasm and are involved in the detection of bacterial PAMPs that enter the mammalian cell. NRLs are especially important in tissues where TLRs are expressed at low levels [29]. This is the case in the epithelial cells of the GI tract where the cells are in constant contact with the microbiota, and the expression of TLRs must be down-regulated in order to avoid over-stimulation. On the other hand, if these epithelial gut cells become infected with invasive bacteria or bacteria interacting directly with the plasma membrane, they will come into contact with NLRs and defence mechanisms can be activated [30]. NLRs are also involved in sensing other endogenous warning signals which will result in the activation of inflammatory signalling pathways, such as nuclear factor-*kappa* B (NF-κB) and mitogen-activated protein kinases (MAPKs). Both NOD1 and NOD2 recognise peptidoglycan moieties found in bacteria. NOD1 can sense peptidoglycan moieties containing meso-diaminopimelic acid, which primarily are associated to gram-negative bacteria. NOD2 senses the muramyl dipeptide motif that can be found in a wider range of bacteria [31,32]. The ability of NRLs to regulate, for example, nuclear factor-*kappa* B (NF-κB) signalling and interleukin-1-*beta* (IL-1β) production, indicates that they are important for the pathogenesis of inflammatory human diseases, such as Crohn’s disease. The role of NLRs in innate immunity and inflammatory diseases has been thoroughly reviewed by Chen *et al.* [33].

NLRs and TLRs interplay in the regulation of the inflammatory response towards bacteria. The expression level of TLRs on the gut epithelium is sophisticated in order to prevent over-stimulation and permanent activation. The GI microbiota can alter this response and the interaction can occur in different ways. The follicle-associated epithelium, which covers Peyer’s patches, is located along the small intestine and is particularly abundant in the ileum. The epithelium harbours shorter villi and contains specialised cells, called microfold cells (M cells). M cells have numerous microfolds on the epithelial side and are specialised in capturing soluble antigens, apoptotic epithelial cells or bacteria from the luminal compartment, and transport them to Peyer’s patches for sampling by dendritic cells or destruction by macrophages [7]. Dendritic cells may present antigen locally to T cells, migrate to T cell zones or to mesenteric lymph nodes, or interact with memory B cells [34]. Both pathogenic and non-pathogenic bacteria can also enter the mucosal tissue through lamina propria associated dendritic cells, which extend their dendrites through epithelial cell tight junctions [6]. Also, the intraepithelial lymphocytes located in the epithelium might recognise microbial antigens [35]. 

In addition to intestinal epithelial cells, the epithelium includes specialised cells such as goblet cells, which secrete the protective mucus layer limiting the contact between bacteria and epithelial cells, and Paneth cells, which reside in the crypts of the small intestine and secrete bactericidal peptides [36]. Secretory IgA is the predominant class of immunoglobulin found in intestinal secretions. It is produced by plasma cells residing in the lamina propria and is transported to the lumen by the polyimmunoglobulin receptor. IgA molecules contribute to specific immunity by capturing antigens, thereby inhibiting mucosal penetration [37].

Inflammation is a consequence of allergy and autoimmune diseases such as arthritis, diabetes type 1, multiple sclerosis and Crohn’s disease, but a low-grade systemic inflammation also characterises the metabolic syndrome and the ageing body. Long-term inflammation increases the risk for heart and cardiovascular diseases, and non-alcoholic fatty liver disease (NAFLD). It also increases the risk of cancer and dementia. Diabetes 2 and obesity are indeed characterised by a low-grade inflammation but it is still unclear if the inflammation is the cause of the condition or just a consequence of it. The bacterial flora (microbiota) of the gut is significant in relation to inflammation, and so favourable influence on the composition of the gut microbiota can be a strategy to mitigate inflammation. Ingesting probiotics (health-beneficial bacteria) can affect the composition of the resident gut microbiota, but probiotics may also have more direct effects on the immune system and the permeability of the mucosa. The better the barrier effect of the mucosa the smaller the risk of translocation of pro-inflammatory components originating from the gut microbiota. 

## 2. Human Gastrointestinal Microbiota

### 2.1. Viable Count, Metagenomics and the Phylogenetic Core

The human GI microbiota starts already in the mouth, which harbours a viable count of 10^8^–10^10^ colony forming units (CFU) of bacteria per g saliva. These bacteria are constantly fed to the GI channel by the swallowing reflex. The numbers are reduced in the stomach (around 10^3^ CFU/g gastric juice), duodenum and jejunum (10^2^–10^4^ CFU/g content), and then increase again in ileum and colon (around 10^10^ CFU/g content and 10^10^–10^12^ CFU/g content, respectively). These bacteria are of different types and, traditionally, attempts to identify them have been done by pure-culture technique, *i.e.*, isolates are cultured at the laboratory and both phenotypic and genotypic characteristics are studied in pure cultures. Current methods are more directed towards direct gene-identification, and mostly towards the 16S ribosomal RNA (rRNA) gene but, lately, “shotgun” Sanger sequencing or massively parallel pyrosequencing have also been used in an attempt to obtain unbiased samples of all genes of a community [38]. The term “metagenomics” is frequently used as a label for studies where more or less all the genetic material is recovered and identified directly from environmental samples [39]. For example, the latter principle was used on faeces of 124 individuals, and each one of the individuals was shown to harbour at least 160 prevalent bacterial species in faeces [40]. Some species are found in many individuals and some are only found in a few. In an attempt to establish the existence of a phylogenetic “core” of the microbiota common for a majority of individuals, Tap *et al.* [41] obtained 10,456 16S rRNA gene sequences by PCR-amplification and cloning from faeces of 17 individuals. 3180 operational taxonomic units (OTUs) were detected, but most of these only appeared in a few individuals, and only 2.1% of the OTUs were present in more than 50% of the faecal samples. On the other hand, most of the OTUs belonged to the phyla *Firmicutes* (about 80%), *Bacteroidetes* (about 20%), *Actinobacteria* (about 3%), *Proteobacteria* (1%) and *Verrumicrobia* (0.1%). Consequently, when bacteria are identified on higher hierarchical levels of taxonomy such as phylum (division) and class, the individual differences between persons appear to be smaller while the differences between habitats within the same individual are more pronounced. For example, there is a significant difference in the composition of the microbiota between the oral cavity and rectum (measured in stool) [42], and between jejunum and colon [43]. Furthermore, the general profile of the GI microbiota of an individual seems to be reasonably stable over time [42]. Frequently dominating genera in the human GI channel are summarised in Table 1.

**Table 1:** Taxa dominating the bacterial microbiota of the GI-tract ^(1)^.

Phyla/Division	Class	Family	Genus	Gram ^(2)^
*Actinobacteria*	*Actinobacteria*	*Micrococcaceae*	*Rothia* *	+
*Actinobacteria*	*Actinobacteria*	*Bifidobacteriaceae*	*Bifidobacterium*	+
**	**	**	**	
*Firmicutes*	*Bacilli*	*Streptoccaceae*	*Streptococcus*	+
*Firmicutes*	*Bacilli*	*Lactobacillaceae*	*Lactobacillus*	+
*Firmicutes*	*Bacilli*	*Enterococcaceae*	*Enterococcus*	+
*Firmicutes*	*Negativicutes*	*Veillonellaceae*	*Veillonella*	(−)
*Firmicutes*	*Negativicutes*	*Veillonellaceae*	*Dialiser*	(−)
*Firmicutes*	*Clostridia*	unclassified *Clostridiales*	*Mogibacterium* *	+
*Firmicutes*	*Clostridia*	*Peptostreptococcaceae*	*Peptostreptococcus* *	+
*Firmicutes*	*Clostridia*	*Lachnospiraceae*	*Coprococcus*	+
*Firmicutes*	*Clostridia*	*Lachnospiraceae*	*Dorea*	+
*Firmicutes*	*Clostridia*	*Lachnospiraceae*	*Roseburia*	(−)
*Firmicutes*	*Clostridia*	*Lachnospiraceae*	*Butyrivibrio*	(−)
*Firmicutes*	*Clostridia*	*Ruminococcaceae*	*Ruminococcus*	+
*Firmicutes*	*Clostridia*	*Ruminococcaceae*	*Faecalibacterium*	+
*Firmicutes*	*Clostridia*	*Ruminococcaceae*	*Anaerotruncus*	+
*Firmicutes*	*Clostridia*	*Ruminococcaceae*	*Subdoligranulum*	+
*Firmicutes*	*Clostridia*	*Clostridiaceae*	*Clostridium*	+
*Firmicutes*	*Clostridia*	*Clostridiaceae*	*Blautia*	+
*Firmicutes*	*Clostridia*	*Eubacteriaceae*	*Eubacterium*	+
*Firmicutes*	*Clostridia*	unclassified	*Collinsella*	+
*Firmicutes*	*Erysipelotrichia*	*Erysipelotrichaceae*	*Holdemania*	+
**	**	**	**	
*Proteobacteria*	*Betaproteobacteria*	*Alcaligenaceae*	*Sutterella*	-
*Proteobacteria*	*Betaproteobacteria*	*Neisseriaceae*	*Neisseria*	-
*Proteobacteria*	*Deltaproteobacteria*	*Desulfovibrionaceae*	*Bilophila*	-
*Proteobacteria*	*Gammaproteobacteria*	*Pasteurellaceae*	*Haemophilus* *	-
*Proteobacteria*	*Gammaproteobacteria*	*Enterobacteriaceae*	*Enterobacter* *	-
*Proteobacteria*	*Gammaproteobacteria*	*Enterobacteriaceae*	*Serratia* *	-
*Proteobacteria*	*Gammaproteobacteria*	*Enterobacteriaceae*	*Escherichia*	-
*Proteobacteria*	*Gammaproteobacteria*	*Enterobacteriaceae*	*Klebsiella*	-
*Proteobacteria*	*Gammaproteobacteria*	*Moraxellaceae*	*Acinetobacter*	-
*Proteobacteria*	*Gammaproteobacteria*	*Pseudomonadaseae*	*Pseudomonas* *	-
*Proteobacteria*	*Gammaproteobacteria*	*Cardiobacteriaceae*	*Cardiobacterium*	-
**	**	**	**	
*Bacteroidetes*	*Bacteroidia*	*Prevotellaceae*	*Prevotella* *	-
*Bacteroidetes*	*Bacteroidia*	*Porphyromonadaceae*	*Porphyromonas* *	-
*Bacteroidetes*	*Bacteroidia*	*Porphyromonadaceae*	*Parabacteroides*	-
*Bacteroidetes*	*Bacteroidia*	*Bacteroidaceae*	*Bacteroides*	-
*Bacteroidetes*	*Bacteroidia*	*Rikenellaceae*	*Alistipes*	
**	**	**	**	
*Fusobacteria*	*Fusobacteria*	*Fusobacteriaceae*	*Fusobacterium*	-
**	**	**	**	
*Spirochaetae*	*Spirochaetes*	*Brachyspiraceae*	*Brachyspira*	-
**	**	**	**	
*Verrucomicrobia*	*Verrucomicrobiae*	*Verrucomicrobiaceae*	*Akkermansia*	-

^(1)^ Genus identification has been made by direct gene identification, mostly of the 16S rRNA gene by cloning and sequencing; ^(2)^ Negative Gram-reaction within parenthesis means that the reaction is negative or variable. It has been shown for *Butyrivibrio fibrisolvens* that the negative gram-reaction is due to a thin cell wall and that the cell wall has Gram-positive characteristics [44]. Presumably this is also the case for the other *Butyrivibrio* spp. and perhaps also for other *Firmicutes* with Gram-negative reaction, *i.e.*, they presumably do not contain lipopolysaccharides (LPS) and are usually associated with a Gram-negative cell wall. * Taxa typically found dominating in the upper GI tract (mouth to jejunum) but mostly much less pronounced in the distal GI tract (ileum to rectum); data from Pettersson *et al.* [45], Wang *et al.* [43], Hayashi *et al.* [46], Bik *et al.* [47], Lazarevic *et al.* [48], Li *et al.* [49], Nasidze *et al.* [50], Turnbaugh *et al.* [51] and Qin *et al.* [40].

### 2.2. Mouth

According to Lazarevic *et al.* [48], dominating phyla in the oral cavity are *Firmicutes*, *Proteobacteria*, *Actinobacteria*, *Fusobacteria*, an uncultured group of 16S rRNA gene sequences labelled TM7 (TM for “Torf, mittlere Schicht” = peat, middle layer) [52] and, to a lesser extent, *Spirochaetes*, while other studies have found *Proteobacteria*, *Firmicutes*, *Actinobacteria*, and *Bacteroidetes* to be the dominant phyla [53]. The most frequently identified genera were *Neisseria* and *Streptococcus*, constituting about 70% of the sequences [48]. However, saliva samples from a larger number of individuals (10 individuals from each of 12 worldwide locations) showed that more than 70% of 16S rRNA gene sequences belonged to the genera *Streptococcus*, *Prevotella*, *Veillonella*, *Neisseria*, *Haemophilus*, *Rothia*, *Porphyromonas*, and *Fusobacterium* [50]. A further 93 genera could be identified (known genera), but a phylogenetic analysis suggested that 64 unknown genera were also present in the saliva samples. The most frequent genus of all in the saliva was *Streptococcus*, which accounted for 23% of the sequences [50].

There is a high bacterial diversity in the mouth and huge differences between people, but mostly there seem to be relatively minor geographic differences [50]. Consequently, there was significantly more variation among sequences from different individuals than among sequences from the same individual, and there was not significantly more variation among individuals from different geographic locations than among individuals from the same location [50]. However, the two genera *Enterobacter* and *Serratia* (both belonging to the family *Enterobacteriaceae*) varied significantly in frequency between locations, e.g., *Enterobacter*, which accounted for 28% of the sequences obtained in samples from Congo, was completely absent in samples from California, China, Germany, Poland, and Turkey. *Serratia* occurred at relatively high frequency in several individuals from Bolivia [50]. 

### 2.3. Stomach

The stomach has always been considered as a relatively harsh environment for bacteria and due to the low viable counts found there, it can always be debated whether the bacteria found are resident or transient (with the exception of *Helicobacter pylori*, the causative agent of gastric ulcers). An adult produces about two litres of gastric juice daily and the pH in lumen is below 2 under fasting conditions, but 5–6 close to the epithelial cells due to the mucus layer. Based on 16S rRNA gene identification, Bik *et al.* [47] found that the dominating phyla on the gastric mucosa were *Proteobacteria*, *Firmicutes*, *Actinobacteria*, *Bacteroidetes*, and *Fusobacteria*, with *Helicobacter* (all sequences were identified as *H. pylori*), *Streptococcus* and *Prevotella* as the most abundant genera. A similar pattern was seen by Li *et al.* [49] who found that the most common phyla were the same as reported by Bik *et al.* [47], except in another order with *Proteobacteria* as the least frequently occurring phylum. Besides the genera *Streptococcus* and *Prevotella*, *Neisseria*, *Haemophilus* and *Porphyromonas* also represented a substantial proportion of the identified clones. These five phyla made up about 70% of the total number of clones [49].

### 2.4. Small Intestine

#### 2.4.1. Jejunum

In jejunum, the mucosal microbiota of a middle-aged, healthy woman from Sweden was strongly dominated by *Firmicutes* (78% of clones), and to a lesser extent by *Proteobacteria* (13% of clones), *Bacteroidetes*, *Fusobacteria* and *Actinobacteria* [43]. Of the clones, 68% were identified as *Streptococcus* (closely resembling *Streptococcus mitis*, *Streptococcus salivarius*, *Streptococcus oralis*, *Streptococcus parasanguinis* and *Streptococcus anginosus*), and 3% were *Gammaproteobacteria* (*Haemophilus*, *Escherichia*, *Acinetobacter* and *Pseudomonas*) [43]. The *Bacteroidetes* clones were most similar to *Prevotella melaninogenica* and *Prevotella loescheii*. Other *Firmicutes* than *Streptococcus* found on the jejunum mucosa were *Veillonella parvula*, *Mogibacterium neglectum* and *Peptostreptococcus anaerobius* [43]. These results can be compared with the microbiota of jejunum content taken at autopsy of three elderly persons from Japan where also *Proteobacteria* and *Firmicutes* strongly dominated, and with smaller proportions of *Actinobacteria* and *Bacteroidetes* [46]. The *Proteobacteria* were mostly *Klebsiella*, and the *Firmicutes* clones were dominated by *Lactobacillus*, and only relatively few clones of *Streptococcus* were found [46].

#### 2.4.2. Ileum

In ileum, the mucosal microbiota of one middle-aged, healthy woman from Sweden was dominated by *Bacteroidetes* (49% of clones) and *Firmicutes* (39%) and, to a lesser extent, *Verrucomicrobia*, *Proteobacteria* and *Fusobacteria* (biopsies from distal ileum) [43]. The *Bacteroidetes* clones were mostly identified as *Bacteroides thetaiotaomicron*, *Bacteroides vulgatus* and *Bacteroides uniformis* while the *Firmicutes* mostly belonged to *Clostridium* clusters XIVa as defined by Collins *et al.* [54] (*Coprococcus catus*, *Dorea formicigenerans*, *Ruminococcus obeum*, *Clostridium symbiosum*, and *Roseburia intestinalis*), IV (*Faecalibacterium prausnitzii*, *Clostridium orbiscindens* and *Dialiser invisus*), IX and XIVb (*Clostridium lactatifermentans*) and, to a lesser extent, *Streptococcus* [43]. These results can be compared with the microbiota of ileum content taken at autopsy of three elderly persons from Japan where no *Bacteroidetetes*, but many *Proteobacteria* (mostly *Klebsiella*), and *Firmicutes* (dominated by *Enterococcus*, *Lactobacillus* and *Streptococcus*) were found [46]. 

### 2.5. Large Intestine

In colon and rectum, the mucosal microbiota of a middle-aged, healthy woman from Sweden was dominated by *Firmicutes* and *Bacteroidetetes*, the former represented by *Clostridium* clusters XIVa as defined by Collins *et al.* [54] (*Eubacterium halii*, *Eubacterium eligens*, *Dorea formicigenerans*, *Ruminococcus lactaris*, *Ruminococcus gnavus*, *Ruminococcus torques*, *Clostridium symbiosum*, *Clostridium boltei* and *Roseburia intestinalis*), IV (*Faecalibacterium prausnitzii* and *Clostridium orbiscindens*), IX (*Dialister invisus*), XIVb (*Clostridium lactatifermentans*), and the latter by *B. vulgatus*, *B. thetaiotaomicron*, *Bacteroides ovatus* and *B. uniformis* [43]. Minor proportions of *Verrucomicrobia*, *Proteobacteria* (*E. coli*, *Acinetobacter johnsonii*, *Sutterella wadsworthensis* and *Neisseria subflava*) and *Fusobacteria* (*Fusobacterium varium*) were also present. These results can be compared with the microbiota of colonic and rectal content taken at autopsy of three elderly persons from Japan where *Firmicutes* strongly dominated, followed by *Proteobacteria* [46]. The former were represented by, for example, subgroups of *Streptococcus salivarius* and *Butyrivibrio fibrisolvens*, and the latter by subgroups of *Klebsiella* and *Escherichia*. The microbiota from sigmoid colon (biopsies) in nine 60-year-old volunteers, without clinical symptoms or medication, showed that a majority of the individuals had a heterogeneous flora, but in one person, 91% of the clones were related to *E. coli* [45]. The microbiota differed widely between individuals with regard to both composition and diversity. The largest number of clones identified close to the level of species for the whole cohort was related to *E. coli*, *Bacteroides vulgatus* and *Ruminicoccus torques*. Most frequently distributed between the volunteers were *Bacteroides uniformis* and *B. vulgatus* (7 out of 9 individuals). *Bacteroides caccae*, *Bacteroides distasonis*, *Bacteroides putredinis*, *B. thetaiotaomicron* and *R. torques* were found in 5 out of 9 individuals. Opportunistic pathogens found in more than one individual were *Bacteroides fragilis*, *Escherichia coli* and *Bilophila wadsworthia*, while *Acinetobacter baumannii*, *Brachyspira aalborgi*, *Cardiobacterium hominis*, *Clostridium perfringens*, *Klebsiella pneumoniae* and *Veillonella parvula* were found in single individuals [45]. In an early report, Hold *et al* [55] investigated the bacterial flora of colonic tissue from three elderly subjects: the flora was dominated by *Bacteroides* and *Firmicutes*, the latter related to either *Clostridium coccoides* (cluster XIVa as defined by Collins *et al.* [54]) or *Clostridium leptum* (cluster IV).

Faeces from nine human, middle-aged subjects were dominated by *Firmicutes* and *Bacteroidetes* and with smaller proportions of *Proteobacteria*, *Actinobacteria*, *Fusobacteria* and *Verrucomicrobia* (sequences of 16S rRNA genes) [56]. Faeces from 156 individuals, 21–32 years old, confirmed the general strong domination of *Firmicutes* and *Bacteroidetes*, and the less pronounced proportions of *Actinobacteria* and *Proteobacteria* [51]. Examples of frequently occurring and dominating species are *B. vulgatus*, *B. uniformis*, *B. thetaiotaomicron*, *Bacteroides ovatus*, *Bacteroides stercoris*, *B. caccae*, *B. putredinis*, *Bacteroides merdae*, *Bacteroides capillosus*, *B. fragilis* and *Parabacteroides distasonis* among the *Bacteroidetes*, and amongst the *Firmicutes*: *Faecalibacterium prausnitzii*, *Eubacterium rectale*, *Eubacterium eligens*, *Eubacterium ventriosum*, *Eubacterium siraeum*, *Ruminococcus obeum*, *R. torques*, *Ruminococcus gnavus*, *Clostridium leptum*, *Clostridium bolteae*, *Clostridium scindens*, *Coprococcus eutactus*, *Dorea longicatena*, and *Anaerotruncus colihominis* [51]. Other frequently found *Firmicutes* in faeces are *Blautia hansenii*, *Clostridium scindens*, *Clostridium asparagiforme*, *Clostridium nexile*, *Ruminococcus gnavus*, *Ruminococcus lactaris*, *Ruminococcus bromii*, *Eubacterium hallii*, *Collinsella aerofaciens*, *Anaerotruncus colihominis*, *Butyrivibrio crossotus*, *Coprococcus eutactus*, *Coprococcus comes*, *Holdemania filiformis*, *Subdoligranulum variabile*, *Dorea formicigenerans*, *Dorea longicatena*, *Streptococcus thermophilus*, *Enterococcus faecalis*. Other frequently found *Bacteroidetes* are *Bacteroides intestinalis*, *Bacteroides pectinophilus*, *Bacteroides finegoldii*, *Bacteroides eggerthii*, *Bacteroides capillosus*, *Bacteroides dorei*, *Bacteriodes xylanisolvens*, *Parabacteroides johnsonii*, *Parabacteroides merdae*, *Roseburia intestinalis* and *Alistipes putredinis* [40]. 

### 2.6. Inflammation Driving Capacity

For some chronic diseases, it has been suggested that the pathologic agent might be the disturbed microbiota rather than a single organism [57], and this presumably means a decreased bacterial diversity and/or different degrees of overgrowth by more aggressive fractions of residential bacteria, *i.e.*, bacteria inducing inflammatory responses by the immune system. A key question is then which bacteria are the most forceful ones in causing inflammation? Naturally, bacterial species known to be pathogenic or opportunistically pathogenic and genera including such species should be more prone to inducing inflammation. Species that are known to include pathogenic or opportunistically pathogenic strains and that also have been found as a substantial part of the gut microbiota of healthy individuals are *E. coli* and *B. fragilis*. Increased proportions of *E. coli* and *B. fragilis* have also been linked to inflammatory bowel diseases (IBD) [58,59,60]. 

Gram-negative bacteria contain lipopolysaccharide (LPS) as the major constituent in the outer leaflet of the outer cell membrane. LPS contains large regions of variable polysaccharide and oligosaccharide regions and a relatively conserved lipid region (lipid A), which is the endotoxic and biologically active moiety responsible for septic shock. The interaction of LPS with macrophages results in the release of pro-inflammatory cytokines such as TNF-alpha, IL-6, and IL-1, and can lead to endotoxic shock, which is an often fatal outcome of sepsis. Although several receptors have been reported to bind to LPS, CD14 has been proven able to mediate these responses *in vivo* [61].

The gram-reaction of different taxa relevant for the GI tract is a factor of importance as Gram-negative bacteria can be expected to contain LPS (Table 1). For example, both the facultatively aerobic *E. coli* and the strictly anaerobic *B. fragilis* contain LPS, but the chemical structures are somewhat different and the mammalian immune system reacts differently towards the different LPS types [62]. The endotoxic activity of LPS of *B. fragilis* is relatively low compared with LPS from *E. coli* and other *Enterobacteriaceae* [63] but, nevertheless, LPS from *Bacteroides* is a potent stimulator of the innate immune system [64]. However, the immune response to LPS can differ between LPS from different species of *Bacteroides* [64,65].

Gram-negatives that typically contaminate foods, and so are ingested on a more or less regular basis, sometimes in high quantities, are mostly *Gammaproteobacteria*, e.g., *Enterobacteriaceaea* and *Pseudomonadaceae*. However, different diet components can also affect the gut microbiota, e.g., a high-fat diet seems to increase the proportion of Gram-negatives in the gut but also increase the leakage of LPS through the intestinal barrier [66]. A theory of how gram-negatives in the gut can affect fattening has been put forward by Cani *et al.* [66]. Diabetes type 2 and obesity are characterised by insulin resistance and low-grade inflammation, and Cani *et al.* [66] showed that LPS in the GI tract was the triggering factor for inflammation and obesity in a mouse model. A high-fat diet increased the LPS concentration in the blood, causing endotoxemia, which induced systemic inflammation, and in turn initiated a process leading to obesity and diabetes in the mouse [67]. It is not known why gram-negative components of the microbiota should be stimulated by a fat-rich diet, or why the barrier function of the mucosa should decrease. However, one speculation could be that a fat-rich diet increases the amount of bile in the gut, and bile has strong antimicrobial effects, but some taxa have higher resistance against bile than others, e.g., *Enterobacteriaceae* and *Bacteroides* are known for their comparably high bile resistance. Furthermore, bile is a powerful detergent which might have effects on the permeability of the mucosa and mediate an increased leakage of LPS.

It should be stressed that it is not only gram-negatives and LPS that can induce inflammation; other cell components and metabolites can be involved, and there are also several gram-positive pathogenic and opportunistic pathogenic bacteria that can induce inflammation [68]. One example of the latter is *Enterococcus*, which is frequently found as a contaminant in foods. 

An attempt to look for correlation between systemic inflammation and faecal microbiota showed that about 9% of the total variability of the microbiota was related to the pro-inflammatory cytokines IL-6 and IL-8 [69]. All taxa that showed a slightly positive correlation with either IL-6 or IL-8 belonged to the phylum *Proteobacteria* [69]. 

It should be borne in mind that different taxa of the microbiota in combination can enhance pathogenic effects. This can be demonstrated in animal models, e.g., in rat models for intra-abdominal sepsis that cause a two-phase disease process consistent with intra-abdominal sepsis in humans, it has been shown that a combination of obligate anaerobes such as *B. fragilis* or *Fusobacterium varium* and facultative aerobes such as *E. coli* or *Enterococcus faecalis* cause early peritonitis and mortality, and abscess development [70]. In this case, *E. coli* was necessary for the mortality, and a combination of *E. coli* and *B. fragilis* was needed for the abscess development [70,71]. Neither *E. coli* nor *B. fragilis* alone provoked abscess formation. Results along the same lines were found in mice infected with *E. coli* and *B. fragilis* in the peritoneal cavity. The co-infection showed an increase in TNF-alpha production in the peritoneal tissues compared with infection by *B. fragilis* alone [72]. KC mRNA in peritoneal tissues was up-regulated after infection with *B. fragilis* which was paralleled by increased KC protein secretion and, after intraperitoneal co-infection with *E. coli* and *B. fragilis*, a synergistic increase in the expression of KC could be noted [72]. *B. fragilis* inhibits the phagocytic killing of *E. coli* [71,73] while *E. coli* inhibits phagocytosis and intracellular killing of *B. fragilis* [74]. Also, *B. fragilis* seems to suppress the *E. coli* associated LPS-induced human endothelial cell adhesiveness for neutrophils [75].

### 2.7. Bacterial Neutralisation of Inflammation

There are fractions of the resident GI microbiota that are less prone to inducing inflammation, and there may even be certain taxa with the ability to counteract inflammation. This seemingly inflammation-suppressing effect can be a result of different actions. The inflammation-suppressing fractions of the microbiota may: (*i*) counteract some of the inflammation-aggravating bacteria, which will decrease the inflammatory tone of the system; (*ii*) improve the barrier effect of the GI mucosa, which allows less inflammation-inducing components in the lumen to translocate out into the body; (*iii*) more directly interact with inflammation-driving components of the immune system. All three actions may be at work simultaneously. 

When the systemic inflammatory tone measured as IL-6 and IL-8 was compared, some members of the *Clostridium* cluster XIVa (as defined by Collins *et al.* [54]) were inversely correlated with systemic inflammation [69]. It has also been shown that a low proportion of *Faecalibacterium prausnitzii* (family *Ruminococcaceae*; *Clostridium* cluster IV or the *Clostridium leptum* group in older vocabulary) on resected ileal mucosa from Crohn’s disease patients is associated with endoscopic recurrence [76]. Furthermore, *F. prausnitzii* was proved to possess anti-inflammatory effects in model systems: secreted metabolites blocked NF-κB activation and IL-8 secretion in Caco-2 cells, and stimulation of peripheral blood mononuclear cells by *F. prausnitzii* led to an anti-inflammatory IL10/IL12 ratio. Oral administration of *F. prausnitzii* also reduced the severity of 2,4,6-trinitrobenzenesulphonic acid colitis in mice [76]. 

The currently most studied inflammation-suppressing taxa of the GI microbiota are certain species/strains of *Lactobacillus* and *Bifidobacterium*, and those are also the fractions that are supported by administering probiotics (living microorganisms that upon ingestion exert health-beneficial effects), or certain dietary fibres that selectively stimulate resident *Lactobacillus* and *Bifidobacterium* (prebiotics). Intestinal exposure to specific bacterial strains may either suppress an undesired immune response, for example allergic and autoimmune reactions, or act in a more generalised immune stimulatory way, associated with adjuvanticity and increased intestinal non-specific IgA secretion [77].

## 3. Probiotics for Humans

### 3.1. Species Used as Probiotics

Originally, probiotics meant organisms or substances that contribute to intestinal microbial balance, in contrast to antibiotics that counteract microbial activity [78]. However a currently widely accepted definition is that “probiotics are live microorganisms which when administrated in adequate amounts confer a health benefit on the host” [79]. In other words, the designation “probiotics” refers to a function, and not to a taxonomic unit. Humans have always ingested bacteria unintentionally together with food. The bacteria could be adverse, but they could also be harmless “dietary bacteria” when fermented foods were consumed. In particular, lactic acid fermented foods such as yoghurt, cheese, sauerkraut, salted gherkins, olives and capers can contain high amounts of live bacteria and often bacteria of the same *Lactobacillus* species that are now used for probiotics. Yoghurt was launched in Paris 1906 with reference to the theories of Metchnikoff [80]. In search of strains with better resistance to the low pH of the stomach and the digestive juices of duodenum, *Lactobacillus acidophilus* was launched in USA in the 1930s, and in Japan during the same period, *Lactobacillus casei* (should probably be *L. paracasei*) started to be used as probiotics. 

Popular probiotic species used commercially include *L. paracasei*, *L. rhamnosus*, *L. acidophilus*, *L. johnsonii*, *L. fermentum*, *L. reuteri*, *L. plantarum*, *Bifidobacterium longum* and *Bifidobacterium animalis*. However, the phylogenetic differences are extremely wide between *Lactobacillus* and *Bifidobacterium* as they belong to different phyla, but there are also great differences between *Lactobacillus* species such as *L. acidophilus*, *L. fermentum*, *L. reuteri* and *L. plantarum*. Even within different strains of the same species, the genomic differences can be considerable, which has been clearly demonstrated for *L. paracasei* [81]. Consequently, with major genetic differences between different probiotics it is also to be expected that the human body will respond differently to different probiotics. This is something that is not always taken into account and it is often neglected in discussions of probiotic effects. Furthermore, it should be stressed that the bacterial species includes considerably genomic heterogenicity. The consensus definition of a bacterial species is that two strains are of the same species if they have a relative ratio of binding of 70% DNA:DNA homology of the genomes at optimal and stringent re-association temperatures (optimal temperature, 25 °C below the melting point of the DNA; stringent temperature, 15 °C below the melting point of the DNA). Consequently, the body can react very differently to different strains of the same species. Unfortunately strain identity is not always given in studies of probiotics administered to humans, e.g., in a failed attempt to improve the clinical outfall in acute pancreatitis where a mixture of strains were given to the patients [82]. The species identity is given in the paper, but no labels on the strains are given. The same is true for a successful attempt to treat acute pancreatitis with a single strain of *L. plantarum* [83]; no strain identity was given. Examples of different human trials with probiotic treatment, and with use of different species/strains are summarised in Table 2. 

**Table 2:** Examples of human trials with probiotics and the strains used for tretament.

Category of subjects	Strains	Major symptom affected	Systemic marker affected	Ref.
Healthy subjects	*L. salivarius* CECT5713	-	NK-cells, monocytes, IgM, IgA, IgG, IL-10	[ 84]
	*L. casei* Shirota	-	NK-cells	[ 85]
	*L. paracasei* Lpc-37, *L. acidophilus* 74-2, *B. animalis* subsp. *lactis* DGCC 420	-	CD57+, phagocytic activity oxidative burst	[ 86]
	*L. acidophilus* 74-2, *B. animalis* subsp. *lactis* DGCC 420	-	phagocytic activity	[ 87]
	*L. rhamnosus* GG	-	Receptors CR1, CR3, FcγRI, IgαR	[ 88]
	*L. plantarum* WCSF1	-	Occluding, ZO-1	[ 89]
Metabolic syndrome and low-grade inflammation	*L. acidophilus* 145, *B. longum* 913	-	HDL-cholesterol	[ 90]
	*L. helveticus* -, *S. cerevisiae* -	Blood pressure	-	[ 91]
	*L. plantarum* 299v	-	total cholesterol, LDL-cholesterol, fibrinogen	[ 92]
	*L. plantarum* 299v	Systolic blood pressure	leptin, fibrinogen, F_2_-isoprostanes, IL-6	[ 93]
	*B. lactis* HN019	-	CD3+, CD4+, CD25+, CD56+, phagocytic activity, tumoricidal activity of NK cells	[ 94]
Non-alcoholic fatty liver disease (NAFLD)	Mixture ^(1)^	-	alanine-aminotransferase (ALAT), γ-glutamyl-transpeptidase, 4-hydroxynonenal, TNF-α	[ 95]
	VSL#3 ^(2)^	-	S-nitrosothiols, malondialdehyde (MDA), 4-hydroxynonenal	[ 96]
Alcohol-related liver injury	*B. bifidum* -, *L. plantarum* 8PA3	-	ALAT, aspartate-aminotransferase (ASAT), gamma glutamyl transpeptidase, lactate dehydrogenase, bilirubin	[ 97]
	*L. casei* Shirota	-	neutrophil phagocytic activity TLR4	[ 98]
Fibrosis, cirrhosis, liver transplantations and minimal hepatic encephalopathy (MHE)	*P. pentoseceus* 5-33:3, *L. mesenteroides* 32-77:1, *L.paracasei* 19, *L. plantarum* 2592	Child-Turcotte-Pugh score	ammonia, endotoxin, bilirubin, ALAT, albumin, prothrombin activity	[ 99]
	*L. acidophilus -*	Clinical status	ammonia	[ 100,101]
	*S. thermophilus*-, *L. bulgaricus* -, *L. acidophilus* -, bifidobacteria -, *L. casei* -	MHE reversal	-	[ 102]
	*L. plantarum* 299	Incidence of postoperative infections	-	[ 103]
	*P.pentosaceus* 5-33:3, *L. mesenteroides* 77:1, *L. paracasei* F19, *L. plantarum* 2362	Incidence of postoperative infections	-	[ 104]
Acute pancreatitis	“Ecologic 641” ^(3)^	-^(4)^	-	[ 82]
Acute pancreatitis	*L. plantarum* -	Clinical outcome	-	[ 83]
Critically ill patients	*L. plantarum* 299v	-	IL-6, intestinal translocation	[ 105]
	*L. plantarum* 299v	-	intestinal translocation, IL-10 white blood cell count, lactate	[ 106]
	VSL#3	-	IgA, IgG	[ 107]
Allergy; infants	*L. acidophilus* LAVRI-A1	-	-	[ 108]
	*L. rhamnosus* GG	Atopic eczema	-	[ 109]
	*B. lactis* Bb-12	SCORAD score	soluble CD4, eosinophilic protein X	[ 110]
	*L. rhamnosus* GG	SCORAD	soluble CD4, eosinophilic protein X	[ 110]
	*L. acidophilus* NCFM, *B. lactis* Bl-04	Nasal symptoms	IgA	[ 111]
	*L. rhamnosus* GG	-	IgA, alpha1-antitrypsin	[ 112]
	mixture ^(5)^	-	IgA	[ 112]
	*L. gasseri* CECT5714, *L. coryniformis* CECT5711	-	IgE, IgA, CD4(+)CD25(+) T regulatory cells, NK-cells	[ 113]
	*B. lactis* Bb12	Body weight	Calprotectin, IgA	[ 114]
Allergy; adults	*L.paracasei* Lpc-37, *L. acidophilus* 74-2, *B. animalis* subsp. *lactis* DGCC 420	-	CD4(+)CD54(+)	[ 86]
	*L. rhamnosus* GG	-	Receptors CR1, CR3, FcγRI, IgαR	[ 88]
Crohn’s disease	*L. rhamnosus* GG	None ^(6)^	-	[ 115]
	*L. rhamnosus* GG	None	-	[ 116]
	*L. rhamnosus* GG	None	-	[ 117]
	*L. rhamnosus* GG	Clinical outcome	-	[ 118]
	*L. rhamnosus* GG	Clinical activity	Intestinal permeability	[ 119]
	*L. johnsonii* LA1	None	-	[ 120]
	*L. johnsonii* LA1	None	-	[ 121]
Ileal pouchitis, ulcerative colitis and colorectal cancer	VSL#3	Disease activity	CD4+CD25^high^ cells, CD4+ LAP+ cells, IL-1β mRNA, Foxp3 mRNA	[ 122]
	VSL#3	Disease activity index, remisson	-	[ 123]
	VSL#3	Remission	-	[ 124]
	VSL#3	Disease activity index, inflammatory bowel disease questionnaire, remission	-	[ 125]
	BIO-THREE ^(7)^	Clinical symptoms, endoscopic findings	-	[ 126]
	*E. coli* Nissle 1917	Clinical symptoms	-	[ 127]
	*L. rhamnosus* GR1, *L. reuteri* RC-14	-	CD4+CD25^high^ cells, IL-12, TNF-α/IL-12-producing monocytes, DCs	[ 128]
	*L. rhamnosus* GG	Remission	-	[ 129]
	*B. breve* Yakult, *B. bifidum* Yakult, *L. acidophilus* -	Clinical activity index, endoscopic activity index	-	[ 130]
	*Bifidobacterium* -, *Lactobacillus* -, *Enterococcus* -	Flare-ups	NF-κB, TNF-α, IL-1β, IL-10	[ 131]
	*Bifidobacterium* -	Postoperative septic complications	SIgA, IgG, IgM, IgA, IL-6, C-reactive protein (CRP)	[ 132]
Radiation-induced enteritis	VSL#3	Diarrhea, bowel movements	-	[ 133]
	*L. rhamnosus* -	Bowel movements, stool consistency	-	[ 134]
	*L. rhamnosus* GG	Diarrhea, abdominal discomfort	-	[ 135]
	*L. acidophilus* -	Diarrhea, flatulence	-	[ 136]
	*L. casei* DN-114 001	Stool consistency	-	[ 137]

^(1)^ Mixture containing *L. acidophilus*, *L. bifidus*, *L. rhamnosus*, *L. plantarum*, *L. salivarius*, *L. bulgaricus*, *L. lactis*, *L. casei*,and *L. breve*; no strain labels are given in the paper; ^(2)^ VSL#3 is a mixture of *L. casei*, *L. plantarum*, *L. acidophilus*, *L. delbrueckii* subsp. *bulgaricus*, *Bifidobacterium longum*, *B. breve*, *B. infantis* and “ *Streptococcus salivarius* subsp. *thermophilus*”; no strain labels are given in the paper; ^(3)^ “Ecologic 641” is a mixture containing *Lactobacillus acidophilus*, *Lactobacillus casei*, *Lactobacillus salivarius*, *Lactococcus lactis*, *Bifidobacterium bifidum*, and *Bifidobacterium lactis*; no species labels are given in the paper; ^(4)^ No effect on occurrence of infectious complications and increased risk of mortality; ^(5)^ Mixture containing *L. rhamnosus* GG ATCC 53103, *L. rhamnosus* LC705, *B. breve* Bbi99, and *Propionibacterium freudenreichii* subsp. s *hermanii* JS 2; ^(6)^ “None” is indicating that no significant effects on symptoms or clinical outcome could be noted; ^(7)^ BIO-THREE is a mixture of *Streptococcus faecalis* T-110, *Clostridium butyricum* TO-A and *Bacillus mesentericus* TO-A.

### 3.2. Immune Modulation

#### 3.2.1. T Regulatory Cells: A Key Factor in Several Dysfunctions

Modification of immune responses in humans is an important potential mechanism by which probiotic bacteria may confer health benefits. Regulatory T cells are involved in the regulation of immune response, maintaining immunological self-tolerance and immune homeostasis, and the control of autoimmunity and cancer surveillance. Consequently, T cells play a key role in autoimmunity, allergy, cancer, infectious disease, and the induction of transplantation tolerance. T cells are characterised by the expression of FoxP3 and additional characteristics include constitutive expression of IL-2 receptor alpha (CD25), the T cell activation marker CTLA-4 and the cell survival factor GITR [138,139].

The capacity of probiotic bacteria to affect regulatory T cells has only been evaluated in a few human trials. How the regulatory T cells function in relation to the subsequent development of an early allergic phenotype, after a probiotic supplementation to infants during their first 6 months of life, has been evaluated but it did not appear to modify the regulatory pathways or the risk of developing atopic dermatitis [108]. However, in patients with ulcerative colitis, different results have been found. In humans, CD4+CD25+ T lymphocytes with regulatory activity reside in the population of CD25+ T lymphocytes with a high expression of CD25 on the cell surface (CD4+CD25^high^) [140]. Patients suffering from inflammatory bowel disease have an increased number of lamina propria CD4+CD25^high^ cells in inflamed tissue compared with control patients, although it is not sufficient to dampen inflammation [141]. Patients undergoing ileal pouch anal anastomosis for UC were randomised in an open-label study of a probiotic mixture of different strains, VSL#3, for 12 months. VSL#3-treated patients showed a significant reduction in pouchitis disease activity index score and a significant increase in the percentage of infiltrating CD4+CD25^high^ and CD4+ LAP-positive cells to the lamina propria, compared with baseline values. Tissue samples revealed a significant reduction in IL-1β mRNA expressions, and a significant increase in Foxp3 mRNA expression. During mild inflammation, this expansion of regulatory cells seems to be adequate to dampen inflammation leading to pouchitis [122].

In an open-label study, 20 patients with IBD (15 with Crohn’s disease and 5 with ulcerative colitis) and 20 healthy subjects consumed probiotic yoghurt containing *L. rhamnosus* GR-1 and *L. reuteri* RC-14 for 30 days. The aim of the study was not to cure IBD or to study the clinical outcomes of the treatment, but to determine whether the consumption induced an anti-inflammatory environment in the patients. After consumption, a significantly increased proportion of CD4+CD25^high^ cells were found in the peripheral blood of IBD patients. Decreased concentrations of IL-12 in serum as well as decreased percentages of TNF-α- and IL-12-producing monocytes and myeloid dendritic cells were also detected. Furthermore it was observed that the production of TNF-α and IL-12 correlated to the number of CD4+CD25^high^ cells in IBD patients. Even if the changes of immunological parameters found in healthy subjects were fewer and more moderate, they were in line with those found in the patients. To verify the influence of the probiotic bacteria, the treatment scheme was repeated with a subpopulation of the same patients after a washout period, using unsupplemented yoghurt. After this consumption, no significant changes could be seen in the percentage of CD4+CD25^high^ cells or percentages of TNF-α- and IL-12-producing cells [128].

Systemic IgA and IgG concentrations have been shown to be increased in intensive care patients suffering from multiple organ dysfunction syndrome given the mixture of probiotic strains, VSL#3, for 7 days [107]. Through production of TGF-β by regulatory T cells in the mucosa, the B cell function can be modulated and antibody class switching may be determined by stimulating switching to IgA [142,143].

#### 3.2.2. Healthy and Allergic Adults

When 40 healthy adults were given *Lactobacillus salivarius* for four weeks, the concentration of NK cells and monocytes increased, together with the plasma levels of immunoglobulins M, A and G, and the regulatory cytokine IL-10 [84]. Also, ingestion of *Lactobacillus casei* strain Shirota for three weeks increased the activity of the NK cells [85]. The relative risk for infection increases with decreasing NK cell activity [144]. The increase in NK cells induced by probiotics could also stimulate a Th1 phenotype with positive effects on allergic patients with Th2 predominance [113].

A mixture of *L. paracasei*, *L. acidophilus* and *B. animalis* subsp. *lactis* was given for eight weeks to adults with atopic dermatitis (AD) and to healthy controls [86]. Major lymphocyte subsets were not affected but the expression of CD57+ (mainly expressed on the natural killer cells) increased significantly in healthy subjects after probiotic intake but was not changed in the AD patients, whereas the expression of CD4(+)CD54(+) decreased significantly in the patients and remained unaffected in the healthy subjects. ICAM-1 (CD54+) is an adhesion molecule that is up-regulated during inflammation, as indicated in the atopic patients. After the probiotic treatment, the phagocytic activity of monocytes and granulocytes and oxidative burst activity was also increased in the healthy controls [86]. The elevated expression of CD57+ in the healthy subjects indicates a stimulation of the immune system, which may decrease the theoretical risk of infections. Increased phagocytic activity in healthy subjects was also found after administration of *L. acidophilus* and *B. animalis* subsp. *lactis*, where the probiotics were able to elevate the percentages of granulocytes and monocytes showing phagocytic activity, but in this case the oxidative burst activity remained unaffected [87].

*L. rhamnosus* strain GG prevented an increased expression of phagocytosis receptors (CR1, CR3, FcγRI and IgαR) in milk-hypersensitive subjects, indicating that the probiotic bacteria had the potential to down-regulate the inflammatory response induced by milk [88]. In the control group consisting of healthy subjects, *L. rhamnosus* GG had an immune-stimulatory effect observed as increased receptor expression after milk consumption containing *L. rhamnosus* GG. It was hypothesised that microbial stimulation by probiotic bacteria may modulate the immune response differently in healthy individuals, where it appears to stimulate a nonspecific immune response to pathogens, while in hypersensitive subjects it down-regulated the inflammatory response [88]. It can be speculated whether the underlying mechanism is associated with an existing difference in composition of the resident microbiota. Depending on the health status of the individual, an aggravating or a suppressing microbiota could be present. The interaction between various immune-competent cells may generate divergent immune-regulatory signals [86].

#### 3.2.3. Allergic Children

The composition of the intestinal microbiota has been implicated in the development of atopic diseases, and in a large prospective birth cohort study the intestinal microbiota of nearly 1000 infants aged one month was examined. The infants were monitored for subsequent development of atopic manifestations and/or sensitisation within the first two years of life. The study demonstrated that the presence of *E. coli* was associated with a higher risk of developing eczema and this risk was increased with increasing numbers of *E. coli* [145]. Furthermore, colonisation with *Clostridium difficile* was associated with a higher risk of developing eczema, recurrent wheeze and allergic sensitisation, and also with a higher risk of a diagnosis of atopic dermatitis [145]. The results indicate that differences in gut microbiota composition precede the development of atopy and since different species were associated with different outcomes, the underlying mechanisms explaining these associations may vary [145]. It has also been seen that low bacterial diversity found in one-week-old infants more frequently gave rise to the diagnosis atopic eczema after 18 months than those with high bacterial diversity [146].

An allergic reaction is the result of an inappropriate immune response triggering inflammation, and several studies have been performed to investigate potential immunoregulatory properties of probiotics in children. Specific probiotic strains have been demonstrated to be effective in prevention of early atopic disease in children at high risk [109], but also as curative of atopic eczema with improvement in skin condition and reductions in serum concentration of soluble CD4 and eosinophilic protein X in urine [110], suggesting mitigated allergic inflammation both locally and systemically. Allergy symptoms from birch pollen in children were assessed by administration of *L. acidophilus* and *B. lactis*. The combined probiotic strains prevented the pollen-induced infiltration of eosinophils into the nasal mucosa, and a trend for reduced nasal symptoms was indicated [111]. Consequently, the results showed that probiotics taken orally affect the inflammatory processes involved in airway allergies. The faecal levels of bifidobacteria, clostridia and *Bacteroides* were reduced at the peak of the birch pollen season. Even faecal IgA was increased in the placebo group during the pollen season, but this increase was prevented by the probiotics [111].

Inflammation in the gut has been shown in children with atopic eczema/dermatitis syndrome (AEDS) and food allergy [147,148]. In a randomised double-blinded manner and concomitant with elimination diet, 230 infants with AEDS and suspected cow’s milk allergy were given either *L. rhamnosus* GG, or a mixture of four probiotic strains (*L. rhamnosus* GG, *L. rhamnosus* LC705, *Bifidobacterium breve* Bbi99, and *Propionibacterium freudenreichii* subsp. s*hermanii*) for four weeks. IgA levels tended to be higher in the probiotic groups than in the placebo group, and alpha1-antitrypsin decreased by administration of *L. rhamnosus* GG, which may indicate that *L. rhamnosus* GG may alleviate intestinal inflammation in infants with AEDS and cow's milk allergy [112].

Compared to a conventional yogurt, a probiotic product containing *Lactobacillus gasseri* and *Lactobacillus coryniformis* enhanced innate and specific immune parameters in allergic children by decreasing the level of IgE in plasma (IgE rise in response to allergens in predisposed atopic subjects), increasing CD4(+)/CD25(+) T regulatory cells as well as natural killer cells [113]. The decrease in IgE was accompanied by a significant increase in mucosal IgA [113], which may be caused by the regulatory T cells. The mucosal immune system contains T cells capable of positively regulating IgA-specific isotype differentiation, thereby allowing for efficient generation of IgA-secreting B cells.

Preterm infants are prone to abnormal bacterial colonisation of the intestine with ensuing adverse health effects. Oral application of *B. lactis* strain Bb12 for 21 days was used in a double blind, placebo-controlled randomised clinical study performed on preterm infants (<37 gestation weeks). In antibiotic-treated infants, *i.e.*, infants that have been subjected to standard antibiotic therapy, probiotic supplementation resulted in a higher body weight compared with placebo. Faecal calprotectin (used as a marker of gastrointestinal inflammation) was lower in the probiotic group, while faecal IgA was higher in this group compared with the placebo group [114]. Probiotics can increase levels of IgA-producing cells in the lamina propria and promote IgA secretion into the luminal mucous layer.

### 3.3. Metabolic Syndrome and Low-Grade Inflammation

The metabolic syndrome is a combination of disorders that increase the risk of developing cardiovascular disease and diabetes. Factors contributing to the syndrome are increased triglycerides in the blood, decreased HDL cholesterol in the blood, increased blood pressure, increasing fasting plasma glucose and central obesity. The metabolic syndrome is characterised by a systemic, low-grade inflammation. LPS leaking out into the body from the gram-negative part of the intestinal microbiota may be the triggering factor for the low-grade inflammation, so probiotics may be a means to improve the gut-barrier and suppress gram-negatives in the GI channel. The ability of many *Lactobacillus* strains to counteract, for example, *E. coli* is well known, and the ability of certain probiotic strains, for example, *L. plantarum* 299v, to mitigate bacterial translocation has been proved in animal models but it has also been tentatively shown in humans [105,106]. Furthermore it has been shown in healthy humans that *L. plantarum* WCSF1 increased the relocation of occludin and ZO-1 into the tight junction area between duodenal epithelial cells [89]. The ability of different *Lactobacillus* strains to improve the barrier effect of the mucosa and suggested mechanisms for this has recently been reviewed by Ahrné and Johansson Hagslätt [149].

In connection to the metabolic syndrome, it must also be mentioned that the GI microbiota of mice seems to be essential for the processing of dietary polysaccharides [150], and in humans it has been shown that the relative proportion of *Bacteroidetes* in comparison with *Firmicutes* is lower in obese individuals than in lean ones; the increased abundance of *Bacteroidetes* correlated with percentage loss of body weight [151]. Furthermore, the proportion of *Bacteroidetes* increased with time in obese individuals put on a low-calorie diet [151]. To certain extent this contradicts the suggestion that the LPS should be the trigger of the metabolic syndrome, as members of the phylum *Firmicuses* do not contain LPS. On the other hand, the genus *Lactobacillus* belongs to *Firmicutes*, and probiotic lactobacilli have been accused of contributing to exaggerated weight-gain [152,153]. The accusation has been turned down most convincingly by Ehrlich [154] and Delzenne and Reid [155]. It must be borne in mind that the phylum *Firmicutes* is a taxon on a high taxonomic hierarchy and includes an extremely wide genomic variation of bacteria, and that loss of weight in mammals also can be an endpoint for ill-health.

Disorders in the lipid metabolism can cause hypertension, and hypertension is often linked to hypercholesterolemia. Yoghurt supplemented with *L. acidophilus* and *B. longum* increased HDL cholesterol [90], and a sour-milk fermented with *Lactobacillus helveticus* together with the yeast *Saccharomyces cerevisiae* decreased the blood pressure in elderly hypertensive subjects [91]. Furthermore, in a small but randomised, placebo-controlled and double blind study on men with slightly elevated cholesterol levels, it was shown that the concentrations of total cholesterol and of LDL cholesterol were decreased after consumption of *L. plantarum* 299v in a beverage containing rosehip and a small quantity of oats (placebo was a similar beverage without probiotics [92]). The fall in cholesterol level was small but statistically significant. Interestingly, the fibrinogen level in serum also decreased (*P* < 0.001), representing a reduction of 13.5% [92]. Fibrinogen is an acute phase protein, a good marker for systemic inflammation and it is also an independent risk factor for coronary artery disease [156].

Smokers are at increased risk of developing systemic inflammation since tobacco smoke triggers the production of free radicals [157,158]. A controlled, randomised, double-blind trial of smokers consuming *L. plantarum* strain 299v for 6 weeks affected systemic parameters, *i.e.*, the systolic blood pressure decreased, and so did the concentration in blood of leptin, fibrinogen, F_2_-isoprostanes (marker for oxidative stress) and the proinflammatory cytokine IL-6 [93].

The ageing process is known to adversely affect the immune system [159,160]. An association between the inflammatory status and the presence of chronic diseases in elderly has been suggested, but also the interaction of an altered microbiota could contribute to maintaining a low-grade, systemic inflammation [161]. In a recent pilot study of elderly persons, the intestinal load of lactobacilli was linked to the count of white blood cells, blood glucose and content of oxidised low-density lipoprotein (ox-LDL), all risk markers in the pathogenesis of inflammation, metabolic syndrome and cardiovascular disease [162].

Thirty healthy elderly volunteers were given a dietary supplement of a probiotic drink containing *Bifidobacterium lactis* for three weeks. The proportion of mononuclear leukocytes staining positively for CD3+ (T lymphocytes), CD4+ (MHC II–restricted T cells), CD25+, and CD56+ (NK cells) as well as the phagocytic capacity of mononuclear and polymorphonuclear phagocytes and the tumoricidal activity of NK cells increased significantly in blood after the probiotic administration. The greatest relative increase in immune function occurred in individuals with poor immune responses before the intervention [94].

### 3.4. Liver Injury

#### 3.4.1. Liver Homeostasis

**Figure 1:**
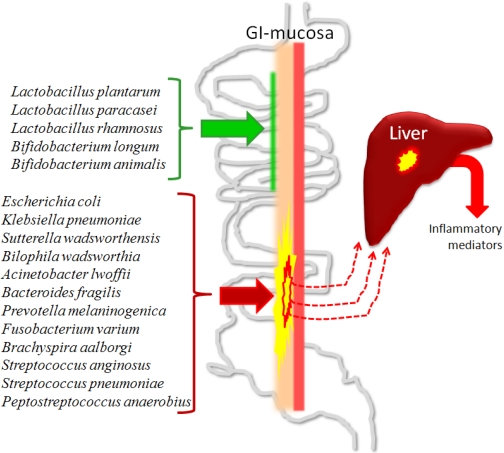
Some examples of bacterial species that are likely to have positive effects on the ecology of the gastro-intestinal (GI) channel (certain strains successfully used as probiotics), and some other examples of species that can occasionally be found as significant parts of resident human microbiota, and are known to possess pathogenic potential (involvement in human infections). The aggressive potential of the adverse species can lead to a weakened barrier effect of the mucosa and leakage of bacterial components that end up in the liver, which will give an inflammatory response. Direct gene identification has shown that the examples of adverse bacteria described form a substantial part of the microbiota in the gastro-intestinal tract of individuals without diagnosed disease [43,45,47,49].

The gut and the liver are closely connected. A well functioning link between the gut and the liver is dependent on both an intact intestine and a liver in balance with respect to immunologic response and metabolism of endogenous and exogenous compounds [163,95]. The intestinal mucosa functions as the local defence barrier that helps to prevent the invasion and systemic spread of bacteria and endotoxins, which are mostly LPS from the cell walls of gram-negative bacteria. However, under certain conditions, intestinal barrier function can be impaired or overwhelmed, allowing bacteria and endotoxins within the GI tract to reach systemic organs and tissues, a process termed bacterial translocation [164]. On the other hand, there is evidence that portal vein endotoxaemia of gut origin in minute amounts is a normal physiological phenomenon [165,166]. During normal conditions, this low-grade endotoxaemia of gut origin is rapidly cleared by the cells of the reticuloendothelial system of the liver [167,168]. Through the portal blood flow draining the GI tract, intestinal bacteria and bacterial products, such as LPS, reach the liver and the parenchymal cells (hepatocytes) and the non-parenchymal cells, encompassing endothelial cells, Kupffer cells, hepatic stellate cells and Pit cells (liver-specific natural killer cells), help to sustain normal physiology and homeostasis, and participate in systemic, as well as in local inflammation and immune response [169]. Some examples of bacterial species that are likely to have positive effects on the ecology of the GI channel (successfully used as probiotics), and some other examples of species that now and then can be found as significant parts of resident human microbiota and known for possessing pathogenic potential are given in Figure 1. 

The aggressive potential of the adverse examples can weaken the barrier effect of the mucosa and allow leakage of bacterial components out into the body. These components will end up in the liver, and the liver will respond with inflammation. Some components of the microbiota will, in contrast, and by different conceivable mechanisms, decrease the leakage of proinflammatory components from the gut, effects so far only proved for certain probiotic strains of the given species. 

The liver is an important site for bacterial phagocytosis and clearance as it contains the largest population of tissue macrophages. Activated Kupffer cells, the resident macrophages of the liver, exposed to pro-inflammatory mediators such as LPS or other bacterial products, are the major source of inflammatory mediators including pro-inflammatory cytokines, chemokines and reactive oxygen/nitrogen species, which contribute to liver injury [170]. However, bacterial particles entering the circulation can also be cleared and detoxified to some extent in the serum by serum proteins such as LPS-binding protein, bactericidal/permeability-increasing protein, and high-density lipoprotein [171]. Through pattern recognition receptors, including Toll-like receptors (TLRs), the innate immune system recognises conserved PAMPs [172]. The healthy liver shows low mRNA levels of TLRs such as TLR1, TLR2, TLR4, TLR6, TLR7, TLR8, TLR9 and TLR10, implying a high tolerance of the liver to TLR ligands from the GI microbiota, to which it is constantly exposed. Signalling through TLRs plays a major role in the physiology and pathophysiology of the liver [173]. 

LPS, membrane components of gram-negative bacteria, are potent activators of innate immune responses through their binding to the TLR4 complex. TLR4 is expressed by Kupffer cells, hepatic stellate cells, hepatocytes, biliary epithelial cells, sinusoidal endothelial cells and hepatic dendritic cells, and are consequently responsive to LPS [173]. There is a positive correlation between liver dysfunction and the occurrence of bacterial translocation, and the clearance of LPS from the circulation is decreased in states of hepatic dysfunction, such as cirrhosis [174].

#### 3.4.2. Fibrosis, Cirrhosis and Minimal Hepatic Encephalopathy

Chronic liver injury is associated with the development of fibrosis, since repeated and continuous hepatocellular damage leads to the activation of hepatic stellate cells and their production of extracellular matrix proteins in the liver. An advanced stage of hepatic fibrosis is cirrhosis, in which functional liver tissue is largely replaced by extracellular matrix and regenerating nodules [175]. The intestinal bacteria seem to be able to induce fibrotic liver disease by means of increased portal delivery of endotoxins, which leads to activation of Kuppfer cells, induction of production of TGF-β and subsequent activation of hepatic stellate cells [176]. The hepatic stellate cells appear to be the main precursors for myofibroblasts in the liver, and are the predominant targets through which TLR4 ligands promote fibrogenesis. 

Minimal hepatic encephalopathy (MHE) is an important disorder in patients with cirrhosis, and a disorder that can seriously impair daily functioning and quality of life. Increased level of ammonia in the blood is most probably a key factor in the pathogenesis [177,178]. Treatment for 30 days with a preparation consisting of four different, non-urease-producing bacterial strains of the species *Pediacoccus pentoseceus*, *Leuconostoc mesenteroides*, *L. paracasei* subsp. *paracasei* and *L. plantarum* along with fermentable fibres was used for the management of MHE [99]. The patients had unusually high faecal loads of *E. coli* and *Staphylococcus* spp., and the probiotic supplementation with the preparation of probiotics and fibres led to reduction of the viable count of *E. coli* and *Staphylococcus*, but also to a reduction of *Fusobacterium* [99]. The treatment led to an increased proportion of non-urease-producing *Lactobacillus* species and decreased ammonia levels in the blood, together with a reduction in the circulating levels of endotoxin. Decreased concentrations of serum bilirubin and ALT (alanine aminotransferase), as well as increases in serum albumin levels and prothrombin activity were found compared to pretreatment values. Also, the Child-Turcotte-Pugh class improved in synbiotic-treated patients [99]. Alterations of the intestinal flora, improvement of the clinical status and lowered blood ammonia levels by the ingestion of probiotics without fibre supplementation has also been shown in previous studies [100,101,102]. 

Early enteral nutrition with solutions containing fibres and probiotics have been suggested to reduce bacterial translocation and minimise the incidence of infections after liver transplantation in cirrhotic patients, and in a prospective, randomised placebo-controlled trial consisting of 95 patients, a marked decreased rate of postoperative infections was found [103,104].

#### 3.4.3. Alcohol-Related Liver Injury

Chronic ethanol consumption causes changes in the liver, including fatty liver, inflammation and cirrhosis [179], and is an established risk factor for the development of hepatocellular carcinoma in patients with liver cirrhosis [180]. Alcoholic steatohepatitis is characterised by infiltration of monocytes, macrophages, neutrophils, and lymphocytes, occurring as a consequence of activation of inflammatory mediators induced by TLR signalling [181,182]. During alcoholic steatohepatitis, serum TNF-α, IL-6, and IL-8 levels are increased and correlate with markers for the acute-phase response, liver function, and clinical outcome [183]. Ultrastructural abnormalities in the epithelial layer of the small intestine and a decreased gut barrier function can be seen in patients with cirrhosis [184,185,186]. Consequently, an impaired gut barrier function might be a cofactor in the progression of chronic liver damage. There is also a strict relationship between altered intestinal permeability and portal hypertension [187]. 

Beneficial effects of probiotics have been reported in an open-label pilot trial, where patients with mild alcoholic hepatitis consumed *Bifidobacterium bifidum* and *L. plantarum* [97]. The treatment resulted in reduction of the levels of alanine aminotransferase (ALT), aspartate aminotransferase (AST), gamma glutamyl transpeptidase, lactate dehydrogenase, and total bilirubin. The microbiota was also affected [97]. In another open-label clinical trial, patients with alcoholic cirrhosis received *L. casei* Shirota three times daily for four weeks. The baseline neutrophil phagocytic capacity in patients was significantly lower compared to healthy controls, but was normalised at the end of the study. TLR2, TLR4 and TLR9 were over-expressed on the surface of neutrophils in patients, but at the end of the study, the expression of TLR4 was also normalised [98].

#### 3.4.4. Non-Alcoholic Fatty Liver Disease (NAFLD)

Non-alcoholic fatty liver disease (NAFLD) comprises a spectrum of diseases ranging from simple steatosis to non-alcoholic steatohepatitis (NASH), fibrosis, and cirrhosis. In its initial phase, the healthy liver becomes steatotic mainly as a consequence of peripheral resistance to insulin, which increases the transport of fatty acids from adipose tissue to the liver. Steatosis renders hepatocytes susceptible to further obstacles. Once steatosis is established, other factors including gut-derived LPS, ethanol, oxidative stress and cytokines aggravate hepatocellular dysfunction, leading to an inflammatory process with hepatocellular degeneration and fibrosis [188]. NAFLD is associated with a number of diseases such as obesity, type 2 diabetes mellitus, hyperlipidaemia, coeliac disease, exposure to different medications and environmental toxins, total parenteral nutrition and surgical procedures (bypass of jejunum or ileum and other operations in the GI tract) [189,190]. The risk of NAFLD was also shown to be more evident in patients with a greater number of adenomatous polyps [191].

An endogenous factor that may contribute to the pathogenesis of NAFLD is the GI microbiota [192]. Hepatic oxidative stress may be increased by enhanced endogenous production of ethanol, and obese female NASH patients present higher levels of breath ethanol [193]. This may be caused by small intestinal bacterial overgrowth, which has been shown in patients with non-alcoholic steatohepatitis [194]. Intestinal bacteria may also affect hepatic oxidative stress through release of LPS, leading to production of inflammatory cytokines by stimulation of luminal epithelial cells and Kupffer cells. Kupffer cells are the main source of TNF-α, a central mediator in the pathogenesis of NASH [195].

It can be speculated whether probiotics might counteract the development of NAFLD by, for example, replacing aggravating bacteria in the GI tract, which in turn can decrease the production of proinflammatory cytokines like TNF-α. An alternative could be that the probiotic bacteria might improve the epithelial barrier function and thereby avoid exposure beyond the normal limit of LPS and ethanol to the liver. However, despite the rationale for the possible therapeutic role of probiotics, no controlled trials have been performed so far in patients with NAFLD/NASH [190]. However, the results achieved from two pilot studies seem promising. A combination of several bacterial strains with probiotic potential improved routine liver damage tests and plasma levels of S-nitrosothiols, malondialdehyde (MDA), 4-hydroxynonenal, alanine transaminase, γ-glutamyltranspeptidase, and TNF-α in NAFLD or NASH patients [95,96].

### 3.5. Ulcerative Colitis, Pouchitis and Colorectal Cancer

Ulcerative colitis (UC) is characterised by periods of remission marked by episodes of clinical relapse caused by acute colonic and/or rectal inflammation. Treatment is primarily aimed at reducing inflammation during relapse and secondarily at prolonging the time spent in remission of clinical symptoms [196]. The histopathological features of UC are characterised by architectural distortion of colonic crypts with frequent depletion of mucin from the goblet cells and diffuse infiltration of lymphocytes and plasma cells. 

During the acute phase of inflammation, macrophages, neutrophils and eosinophils infiltrate the lamina propria of the colonic mucosa. Aggregating neutrophils, especially near the crypts, lead to the formation of abscesses [197]. Activated dendritic cells (DCs) and macrophages secrete cytokines that trigger and differentiate T cells, and activate the adaptive immune response. Increased populations of CD4-positive and CD8-positive cells have been found in the colonic lamina propria of patients with active UC [198]. Upon antigenic stimulation, naive CD4+ T cells are activated, expand and differentiate into different effector subsets of cells (Th1, Th2 and Th17), that are characteristic for the production of distinct cytokines and effector functions [199]. In both UC and Crohn’s disease, polarised immune activity towards Th1 (marked by up-regulation of TNF-α, IL-1β, IFN-γ, IL-6) and Th17 (marked by IL-17 secretion) response is reported, while UC appears to exhibit an added contribution of Th2 responses (characterised by secretion of IL-4, IL-5, and IL-13) [200]. Cytokines, such as IFN-γ and TNF-α, increase the expressions of TLR4 in intestinal epithelial cells, which results in increased LPS responsiveness [201]. During UC, the expression of TLR4 is increased on mucosal DCs as well as on intestinal epithelial cells in inflamed and non-inflamed mucosa throughout the colon and terminal ileum [202,203]. The CD4+ T cell phenotype expressing CD25^high^ and fork-head box protein 3 (FoxP3) has been recognised as the functional representative of regulatory T cells (Treg). The Treg is known to down-regulate immune responses to both foreign and self-antigens [204] and a significant number of T-regulatory cells can be found in the inflamed intestine. Their ability to overcome the inflammatory response is hypothesised to be a major reason for remission, so is a major goal of therapies aimed at enabling the regulatory functions of these naturally immunosuppressive cells [199].

UC patients seem to have higher numbers of bacteria associated to the mucosa than healthy subjects, and the difference may reflect the altered nature of the mucus, which appears to be thinner and less sulphated than that of healthy subjects [205,206]. A thin mucus layer containing larger than normal numbers of bacteria might facilitate contact between bacterial antigens and the mucosal immune system.

The intestinal microbiota in patients with active UC has been shown to be less diverse than in healthy subjects [207]. It is not clear whether endogenous intestinal bacteria and/or specific bacterial pathogens are directly or indirectly involved in the initiation and/or maintenance of UC. Neither is it known which bacterial components or antigens can be responsible for the unrestrained inflammatory response. The colonic surface and the inflamed area of UC patients are colonised by a wide variety of organisms [58]. The *Clostridium histolyticum*/*Clostridium lituseburense* group made up 21% of the microbiota in UC specimens, while these organisms were not found in controls. These phylogenetic groups contain mainly clostridia belonging to clusters I and II, and part of cluster XI of Collins [54], species such as *C. histolyticum*, *Clostridium beijerinckii*, *Clostridium perfringens*, *Clostridium botulinum*, *Clostridium intestinalis or Clostridium lituseburense*, *C. difficile*, *Clostridium bifermentans*. Several of these organisms may be pathogenic. *Enterobacteriaceae* have also been considered as being involved in the pathogenesis of UC, owing to the ability to adhere to the intestinal mucosa and to produce enterotoxins [208]. *E. coli* and *Klebsiella* accounted for 25% of the mucosa-associated and 20% of the mucosa penetrating bacteria [58]. High proportions of *Enterobacteriaceae* and *B. fragilis*, together with a substantial presence of *Pseudomonas aeruginosa* were found on the inflamed colonic mucosa taken during surgery from a 12-year-old girl suffering from UC [60]. Sulphate-reducing bacteria have received attention due to their ability to reduce sulphate to sulphide, a by-product of their respiration. Hydrogen sulphide is freely permeable to cell membranes and inhibits butyrate oxidation in colonocytes [209], and hydrogen sulphide has been implicated in the pathogenesis of UC [210]. 

The number of lactobacilli seems to be relatively low in active UC [211]. However, lactobacilli were predominantly detected in inactive patients, and were suggested to have a role in the induction of remission [212]. It has been hypothesised that the changing condition in the intestine may influence the amount as well as the type of *Lactobacillus* [213,214]. 

The use of probiotics for patients suffering from UC has gained attention, and studies to verify the efficiency have been performed for both intervention and maintenance therapy. A meta-analysis to evaluate the induction of remission and maintenance of probiotic therapy was carried out by Sang *et al.* [215] on thirteen randomised, controlled studies. It was concluded that probiotics were more effective than placebo in maintaining remission [215]. 

The probiotic mixture, VSL#3 for treatment of mild-to-moderate active UC, was analysed by Sood *et al.* [123]. Six weeks of probiotic treatment resulted in a significantly higher percentage of patients with more than 50% improvement in UC Disease Activity Index score, and after 12 weeks, significantly more patients achieved remission [123]. 

Several species and strains of bacteria with claimed probiotic potential have been used in clinical trials, e.g., *E. coli* Nissle [127], a mixture of *L. casei*, *L. plantarum*, *L. acidophilus*, *L. delbrueckii* subsp. *bulgaricus*, *Bifidobacterium longum*, *Bifidobacterium breve*, *Bifidobacterium infantis* and *Streptococcus salivarius* subsp. *thermophilus* (VSL#3) [122,123,124], a mixture of *Streptococcus faecalis*, *Clostridium butyricum*, and *Bacillus mesentericus* (BIO-THREE) [126], a mixture of *L. rhamnosus* and *L. reuteri* [128], *L. rhamnosus* GG [129], *B. breve* strain Yakult, *B. bifidum* strain Yakult and *L. acidophilus* [130]. Probiotics have shown effects in treatment of active mild-to-moderate UC by decreasing clinical activity index, preventing relapse, and induction of remission [123,124,126,127,128,129,130]. Also, improvements of histological scores and increases in faecal butyrate, propionate and short-chain fatty acid concentrations have been registered [130]. Consumption of probiotics by UC patients prevented flare-ups and induced depressed activation of the transcriptional factor NF-κB, decreased expressions of TNF-α and IL-1β, while the expression of IL-10 was elevated [131]. The percentage of CD4+CD25^high^ cells increased in IBD patients after ingestion of a probiotic yogurt [128]. Not much data is available on how probiotics might alter the composition of the resident gut microbiota but it seems that some probiotics can increase the load of lactobacilli and/or bifidobacteria [126,131]. 

Ileal pouch-anal anastomosis is a surgical procedure for management of UC by making an ileal reservoir, a pouch. Unfortunately, a complication frequently occurring is inflammation in the pouch, pouchitis [216]. A meta-analysis of five randomised, placebo-controlled clinical trials indicated an advantage of probiotic administration in the treatment of pouchitis [217]. Pouchitis disease activity index scores, as well as the inflammatory bowel disease questionnaire score, were improved and remission maintenance fulfilled [122,125]. Furthermore, Pronio *et al.* [122] found increased percentages CD4+CD25^high^ cells, CD4+ LAP-positive cells (latency-associated peptide) and a significant reduction in IL-1β mRNA expression in mucosal samples. Since an increase in Foxp3 mRNA expression was also found in mucosal biopsis, this indicates higher numbers of regulatory T cells [122].

The pouch microbiota after probiotic treatment indicated higher bacterial diversity and lower fungal diversity during remission induced by probiotic consumption [218]. The opposite was found for control patients developing pouchitis. A recolonisation and diversification of the lactobacilli and bifidobacteria was shown at remission [218]. 

A variety of hepatobiliary abnormalities have been described in patients with ulcerative colitis, including fatty changes, cholelithiasis, pericholangitis, primary sclerosing cholangitis, cirrhosis, chronic active hepatitis, amyloidosis, and bile duct cancer, with primary sclerosing cholangitis being the most common form [219]. Because gut-derived components are easily accessible to the liver via the portal vein, it is suggested that increases in the permeability of the intestinal epithelium during inflammation allow bacterial antigens and toxins to enter the lamina propria and cause an inflammatory reaction when the bacterial products reach the liver [220]. However, no clinical trials on the effect of probiotics on ulcerative colitis-associated liver injuries seem to have been done. 

Patients with ulcerative colitis also represent a risk group for developing colorectal cancer (CRC). The two most important risk factors seem to be the duration and extent of the disease but the severity of inflammation has also been shown to correlate with an increased frequency of dysplasia and therefore a greater CRC risk [221]. Probiotics have been given preoperatively and postoperatively to CRC patients in order to reduce intestinal pathogens and to modulate immune response. A mixture of *B. longum* and *Lactobacillus johnsonii* in a dose of 10^9^ CFU decreased the concentration of *Enterobacteriacae* in faeces while a dose of 10^7^ CFU failed to do so [222]. The same trend was found for enterococci [222]. The ratio of *Bifidobacterium*/*E. coli* increased in patients given probiotics with enteral nutrition before colorectal cancer resection [132]. Both preoperative and postoperative ratios were significantly lower in the control group. Nine days after surgery, faecal concentration of SIgA increased, but serum IgG, IgM, IgA, IL-6, CRP concentrations decreased. Furthermore, the probiotic treatment also caused less postoperative septic complications [132].

### 3.6. Crohn’s Disease

Similar symptoms may appear during Crohn’s disease (CD) and UC that can give rise to diagnostic difficulties [223]. However, some specific characteristics reflect the different conditions. CD can affect any part of the gastrointestinal tract and the inflammation is transmural and influences the whole intestinal wall [224] while UC mostly affects the superficial lining mucosa in colon and rectum. UC usually begins in the rectum and extends upwards through colon and rarely affects the small intestine [225]. The inflammation in UC has a continuous distribution while CD has a patchy pattern [224]. Analysis of bacterial 16S rRNA genes revealed no significant differences in mucosal bacterial composition between CD and UC patients [226], but a trend towards a larger reduction of diversity was found in UC patients but it was not significant compared to CD patients [226]. Also, a trend has been seen towards a predominance of *Bacteroides* and an increase of mucosal bacteria in CD patients [227].

The efficacy of probiotics for induction of remission in Crohn’s disease was evaluated by Butterworth *et al.* [228] through data bases and register-searching of randomised controlled clinical trials. Twelve potentially relevant studies were identified but eleven were not considered to fit the stated criteria. One study fulfilled the inclusion criteria but it only included 11 patients with moderate- to active-Crohn’s disease [115]. The patients received *L. rhamnosus* GG for six months and they were also given antibiotics one week before the probiotic/placebo treatment was initiated. Sustained remission was the principal endpoint but no significant difference in median time to relapse was observed between placebo and treatment group [115]. 

Children with CD in remission were given *L. rhamnosus* GG in addition to standard therapy in order to try to prolong this state [116]. However, no prolonged remission was obtained [116]. Ineffectiveness of remaining remission by supplementation of probiotics was also found by administration of *L. johnsonii* LA1 after surgical resection [120,121] or by administration of *L. rhamnosus* GG to adults [117]. In contrast to these negative results, preliminary data on four paediatric patients showed significant improvement by administration of *L. rhamnosus* GG [118]. Another small prospective study was performed on four children with Crohn´s disease. The patients were given *L. rhamnosus* GG, and a significant improvement in clinical activity and improved barrier function of the intestine was found [119]. These small pilot studies are needed in order to find an efficient probiotic strain and to provide a base for the estimation of power, in order to be able to include a reasonable number of patients in an extended study. This extended, hypothetical study ought to be a blinded placebo-controlled study, running over a time period clinically relevant for the disease, *i.e.*, the study will be costly and involve serious ethical considerations as a number of patients in such a study will receive a product with no therapeutic effects, and preferably the patients should give up other therapies during the study period. Consequently, in this case, the probiotics must be regarded as a medical drug and not as a supplement of functional foods. If the intention is to evaluate the probiotic effect for a certain bacterial strain intended as ingredient in functional foods, patients with Crohn’s disease hardly form the best test group.

### 3.7. Radiation-Induced Enteritis

Intestinal injury from radiotherapy of pelvic malignancies is clinically important, as enteritis symptoms commonly occur and there are few therapeutic options. Moreover, it has been suggested that protection from injury of normal tissues may provide an increase in tumour control, by allowing an increase in the radiation dose [229,230]. Since the GI mucosa contains sensitive regenerative epithelium susceptible to the toxic effects of ionising radiation, injury to the small and large intestine is among the most significant complications encountered in patients receiving radiation directed at the abdominal or pelvic cavity [231,232]. 

The radiation dose that can be applied in clinical practice is usually limited by the need to restrict the number and severity of side effects in normal tissues surrounding a tumour, which are unavoidably exposed to radiation [233]. Acute radiation enteritis is a potentially serious complication of radiation therapy. Histologically detectable alterations of the intestinal mucosa, like protein and fibrin precipitation, inflammation and oedema of the bowel wall, can be found several days after radiation [234,235]. The villous height and number decreases. The affected functioning of the bowel mucosa leads to the loss of proteins, electrolytes and water. Due to the reduced intestinal surface, conjugated bile salts are not reabsorbed in the small intestine and enter the colon. Local bacterial flora deconjugates the bile salts leading to chologenic diarrhoea [236,237]. 

An early inflammatory response, beginning a few hours after irradiation, is characterised by leucocyte infiltration into the irradiated organs. Radiation activates various cellular signalling pathways that lead to expression and activation of pro-inflammatory and pro-fibrotic cytokines, vascular injury and activation of the coagulation cascade. Certain mucosal cytokines are activated and the levels of TNF-α, IL-1β, IL-2, IL-6, and IL-8 are significantly higher [238].

Radiation influences and disturbs the mucosal microbiota, leading to a translocation of microorganisms or microbial products through the mucosa into the blood circulation [239]. Mucosal permeability of irradiated colon of patients treated for rectal cancer can be expected to be increased. This difference may be attributed to the mucosal atrophy observed in the irradiated patients and may result in an increased risk of radiation enteritis [232]. Translocation of pathogenic organisms through the intestinal wall into the bloodstream, the peritoneal cavity and abdominal organs is a well-recognised cause of supervening sepsis and life-threatening complications in critically ill patients [240].

Clinical trials have implicated probiotic therapy as beneficial against radiation-induced diarrhoea. A double-blind, placebo-controlled clinical trial including almost 500 patients who underwent adjuvant postoperative radiation therapy after surgery for sigmoid, rectal, or cervical cancer has been performed [133]. The patients were assigned to either a probiotic preparation (VSL#3) or placebo treatment, starting from the first day of radiation therapy. The incidence and severity of radiation-induced diarrhoea, the daily numbers of bowel movements, and the use of loperamide were all reduced [133]. Improved stool consistency and reduced number of bowel movements, less abdominal discomfort, and fewer chemotherapy-dose reductions due to toxicity have also been found by the use of *L. rhamnosus* [134,135]. Administration of *L. acidophilus* during irradiation of the pelvic area because of gynaecological malignancies also appeared to prevent radiotherapy-associated diarrhoea [136]. Flatulence was increased though, probably due to lactulose given as substrate for the bacteria [136]. In contrast, it was found for gynaecological malignancies that the incidence of radiation-induced diarrhoea was not reduced by the use of *L. casei* [137]. However, a significant effect on stool consistency was recorded [137]. 

## 4. Conclusions

For most of the examples of probiotic applications mentioned in the present review there is a large amount of evidence on effects and mechanisms in experimental animal models, but human studies in most cases are still relatively rare. The present review has only dealt with observations in humans, and it is obvious that more clinical trials are needed to examine and verify the anti-inflammatory effects of probiotics in regulating systemic inflammation and local mucosal inflammation, and immune-regulation of other dysfunctional immune reactions leading to, for example, allergy and autoimmune diseases. On the other hand, it is also obvious that the resident microbiota from mouth to rectum is an important factor for homeostasis and for the patho-physiological course of events, and that probiotics are promising means of intervention. Popular species for use as probiotics are *L. paracasei*, *L. rhamnosus*, *L. acidophilus*, *L. johnsonii*, *L. fermentum*, *L. reuteri*, *L. plantarum*, *B. longum* and *B. animalis*. However, the phylogenetic differences between these taxa can be huge. There are extremely large variations between *Lactobacillus* and *Bifidobacterium*, but the phylogenetical differences are also substantial between many of the different *Lactobacillus* spp., for example between *L. acidophilus*, *L. fermentum*, *L. reuteri* and *L. plantarum*. Even within different strains of the same species, the genomic differences can be considerable. Consequently, when the major genomic differences between different types of probiotics are taken into account, it is to be expected that the human body can respond differently to the different species and strains. This fact does not always seem have been considered when testing probiotics in humans, and the often huge genomic differences and resultant differences in phenotype is neglected in the discussion of the outcome of human trials with probiotics.

From scientific and clinical perspectives it is of utmost importance to choose good endpoints in human trials with probiotics, *i.e.*, endpoints that beyond dispute show improvement in health status. Examples of such endpoints can be the blood pressure in subjects suffering from the metabolic syndrome, or decrease in liver fat in subjects with fatty liver. On the other hand, these types of hard endpoint may be regarded as too clinical by the legislative authorities if the study is to be used for claiming health benefits for foods or food supplements. In this realm, biological markers for health seem to be more acceptable. The problem here is to find good markers, e.g., immunological markers. An obstacle is that the immune system is a double-edged sword—in one setting, inflammation is required and an increased concentration of inflammatory markers is a sign of a properly acting immune defence, but in another setting inflammation is something negative and increased levels of inflammatory markers indicate a dysfunction in the body. Furthermore, the concentrations of immunological markers change with time—they interact with each other and they mostly have multiple functions. On the basis of Table 2, IL-6, IL-10 (ought to be supplemented with IL-12) and IgA are immunological markers that seem to have been affected in more than one study. However, the markers affected and considered relevant for systemic inflammation vary between the individual studies and between different categories of dysfunctions. A suggestion for future studies on systemic inflammation is to include, as a minimum, measurements of C-reactive protein (CRP), fibrinogen and IL-6, combined with the liver-function markers alanine-aminotransferase (ALAT) and aspartate-aminotransferase (ASAT). These are all markers that frequently are used in clinical settings, and they are reasonably robust. Also, highly relevant are different categories of white blood cells and certain key-receptors for inflammation, e.g., Foxp3 (regulatory T cells), Cd11b and CD11c (macrophages and dendritic cells) and unravelling different colonic macrophage and dendritic cell populations and their functions is of high interest. Furthermore, their TLR2 and TLR4 expression, but also the expression on epithelial cells is an option for clarifying bacterial stimulation. Other useful markers for the inflammatory status of the intestinal mucosa are calprotectin, IgA, myeloperoxidase (MPO), and an incontrovertible end-point is a histopathological evaluation. 
